# Micro-RNA-215 and -375 regulate thymidylate synthase protein expression in pleural mesothelioma and mediate epithelial to mesenchymal transition

**DOI:** 10.1007/s00428-022-03321-8

**Published:** 2022-04-24

**Authors:** Francesca Napoli, Ida Rapa, Stefania Izzo, Angelica Rigutto, Roberta Libener, Chiara Riganti, Paolo Bironzo, Riccardo Taulli, Mauro Papotti, Marco Volante, Giorgio Scagliotti, Luisella Righi

**Affiliations:** 1 Department of Oncology, University of Turin, San Luigi Hospital, Regione Gonzole 10, Orbassano Turin, Italy; 2 Pathology Unit, San Luigi Hospital, Orbassano, Turin, Italy; 3 Present Address: Department of Medical Oncology and Hematology, University Hospital of Zurich, Zurich, Switzerland; 4 Pathology Division, Saints Antonio and Biagio Hospital, Alessandria, Italy; 5 Interdepartmental Research Center of Molecular Biotechnology, University of Turin, Turin, Italy; 6 Interdepartmental Centre for Studies On Asbestos and Other Toxic Particulates, University of Turin, Turin, Italy; 7 Center for Experimental Research and Medical Studies (CeRMS), City of Health and Science University Hospital, Turin, Italy; 8 Pathology Unit, City of Health and Science University Hospital, Turin, Italy

**Keywords:** Pleural mesothelioma, Micro-RNA, Thymidylate synthase, Tissue expression, Epithelial to mesenchymal transition, Cell lines

## Abstract

The standard front-line treatment for pleural mesothelioma (PM) is pemetrexed-based chemotherapy, whose major target is thymidylate synthase (TS). In several cancer models, miR-215 and miR-375 have been shown to target TS, while information on these miRNAs in PM are still limited although suggest their role in epithelial to mesenchymal transition. Seventy-one consecutive PM tissues (4 biphasic, 7 sarcomatoid, and 60 epithelioid types) and 16 commercial and patient-derived PM cell lines were screened for TS, miR-215, and miR-375 expression. REN and 570B cells were selected for miR-215 and miR-375 transient transfections to test TS modulation. ZEB1 protein expression in tumor samples was also tested. Moreover, genetic profile was investigated by means of BAP1 and p53 immunohistochemistry. Expression of both miR-215 and miR-375 was significantly higher in epithelioid histotype. Furthermore, inverse correlation between TS protein and both miR-215 and miR-375 expression was found. Efficiently transfected REN and 570B cell lines overexpressing miR-215 and miR-375 showed decreased TS protein levels. Epithelioid PM with a mesenchymal component highlighted by reticulin stain showed significantly higher TS and ZEB1 protein and lower miRNA expression. A better survival was recorded for BAP1 lost/TS low cases. Our data indicate that miR-215 and miR-375 are involved in TS regulation as well as in epithelial-to-mesenchymal transition in PM.

**Supplementary Information:**

The online version contains supplementary material available at 10.1007/s00428-022-03321-8.

## Introduction

Pleural mesothelioma (PM) is a rare, asbestos-related, relatively chemo-resistant tumor that arises from the mesothelial pleural surface. PM is more common in males and the highest incidence is reported in the sixth and seventh decade of life. The most common PM histological subtype is the epithelioid one, followed by biphasic and sarcomatoid subtypes. Patients generally have poor prognosis, strongly related to histology and to the stage of the disease at diagnosis, with median survival for treated patients from 6 to 18 months for epithelioid and much lower survival rates for the sarcomatoid histotype [1]. Chemotherapy with pemetrexed (PEM) and platinum is the standard of care with improvement in overall survival of roughly three months, compared to single cisplatin agent [2–4].

PEM as a multi-targeted anti-metabolite inhibits multiple molecules of the folate metabolic pathway, especially thymidylate synthase (TS), an enzyme essential for DNA synthesis and repair [5]. TS is overexpressed in various cancer types and is associated with metastatic spread and reduced overall survival. Moreover, TS expression levels are upregulated following treatment with chemotherapy, including agents that inhibit its activity. In several studies, high levels of this enzyme seem to be correlated to reduced PEM efficacy in different tumor types, including mesothelioma, colon, lung, and breast carcinomas [6–11].

Genetic studies on PM reported a low prevalence of oncogene driver mutations and low tumor mutational burden, and the vast majority of recurrent mutations predominantly result in loss-of-function of tumor suppressors, including BAP1, TP53, CDKN2A, NF2, and LATS2 [12, 13]. The low frequency of genomic events in PM has moved the interest on epigenetic regulation of PM growth and progression with possible integration among different mechanisms [14].

Micro-RNAs (miRNAs) are a class of small (about 18–22 nucleotide long) non-coding RNAs that function in post-transcriptional regulation of gene expression [15, 16]. Micro-RNAs are expressed in physiological conditions in a cell- and tissue-specific-manner, but their aberrant expression in tumor tissues is associated with tumor-specific characteristics, thus supporting their potential role as diagnostic, prognostic or predictive biomarkers [17, 18]. Recently, a set of significantly downregulated (miR-874, miR-31, miR-203, miR-200a, miR-143, miR-200c, and miR200b) and upregulated (miR-139-5p, miR-210, miR-944, and miR-320) miRNAs were found in pleural effusion from PM patients [19] compared to other diseases (adenocarcinoma and benign pleural disease) and more recently several studies proposed microRNA as potential therapeutic target for resistant PMs [20–22]. In this context, miR-215 and miR-375 have been already reported as strong modulators of TS in different cancer cells [23], but no data are available about their role on targeting TS in PM. Only miR-215-5p was reported to interact with MDM2-p53 cell signaling and to be associated with poor prognosis in PM [24]. Furthermore, miR-215 and miR-375 are also known to be involved in epithelial-to-mesenchymal transition (EMT) [25–27] and their action was connected, among the others molecules, to ZEB1 (Zinc Finger E-Box Binding Homeobox 1) activity [28, 29]. EMT is both a physiological and pathological process related to embryonic developments [30, 31] as well as to wound healing in fibrotic tissues, tumor development, invasion, and metastatization [32]. In PM disease, the concomitant presence of epithelioid and sarcomatoid histological features suggests a distinct role of epithelial-mesenchymal transition in PM via ZEB1, as already reported [33].

Based on these data, the aims of our study were (i) to investigate miR-215 and miR-375 expression in PM patient tissues in correlation with TS protein and mRNA levels; (ii) to test the possibility of modulating TS levels in PM cell lines by miRNA transfection; and (iii) to explore a potential role of these two miRNAs in regulating EMT in PM.

## Methods

### Patient information

Consecutive tumoral pleural biopsy specimens with left-over formalin-fixed paraffin embedded (FFPE) material were collected from the pathology files of San Luigi Hospital, Orbassano, Turin, from 2013 to 2016. Before starting the study, all cases were anonymized by a pathology staff member not involved in the project and only coded data were used throughout. From a representative paraffin block, tumor areas were selected by a pathologist and microdissected for RNA extraction. Taking into account the retrospective nature of the research protocol and that it had no impact at all on patients’ care, no specific written informed consent was required.

### Cell line cultures

Nine commercially available PM cell lines (H2052, H226, H2452, MPP89, MSTO, MERO-14, REN, SDM103T2, ZL34) were obtained from ATCC (Manassan, VA) while seven primary PM cell lines (404B, 487B, 682B, 672B, 353B, 570B, 421B) were obtained from the Biobank of Saints Antonio and Biagio General Hospital, Alessandria, Italy. Of these, 404B, 487B, 682B, H2052, H2452, H226, REN, and MPP89 were epithelioid-derived; 672B, 421B MSTO, and SDM103T2 were biphasic-derived, and 353B and 570B were sarcomatoid-derived PM cell lines. MERO-14 and ZL34 histotype derivation was not available. All cell lines were maintained in 5% CO2 and 37 °C. MERO-14, ZL34, SDM103T2 together with all primary cell lines were cultured in DMEM/F12; REN, H2052, MSTO, H226, and H2452 cell lines were cultured in RPMI medium; MPP89 cells were cultured in DMEM. All the medium were supplemented with 1% L-glutamine, 1% penicillin (25 U/ml), streptomycin (25 µg/ml) and 10% fetal bovine serum (all from Sigma-Aldrich, St.Louis, MO, USA). Cell line characteristics were summarized in the Supplementary Table 1.

### RNA extraction and real-time PCR

MiRNeasy FFPE extraction kit (QIAGEN, Hilden, Germany) was used for RNA extraction from FFPE specimens, whereas miRNeasy mini kit (QIAGEN) was used for cell lines, both according to the manufacturer’s protocols. The concentration and the purity of RNA samples were assessed using the BioPhotometer (Eppendorf, Hamburg, Germany). A total of 40 ng (for patient specimens) and 10 ng (for cell lines) of RNA was reverse transcribed. The temperature protocol used for RT was as follows: 16 ˚C for 30 min, 42 ˚C for 30 min, 85 ˚C for 5 min. The TS cDNA was synthesized by M-MLV reverse transcriptase (Invitrogen, Carlsbad, California, US) using random primers, according to the manufacturer’s protocol. MiRNA expression was assessed by means of quantitative real time PCR using specific TaqMan MicroRNA Assays (Applied Biosystem, Foster City, California, US) and Taqman Universal Mastermix II, with UNG (Applied Biosystem). RNU6B (cod.001093) TaqMan MicroRNA Assay (Applied Biosystem) was used for miRNA expression normalization. SensiFAST Probe Hi-ROX Mix (Bioline) and specific TaqMan Gene Expression assay (Applied Biosystem) were used for the TS gene (TYMS) detection (cod. Hs00426586_m1) and for ACTB (cod. Hs03023943_g1), used as internal reference gene. A mixture containing human total RNA (Stratagene) was used as a control calibrator. Thermocycling conditions were as follows: 50 °C for 2 min, 95 ˚C for 10 min followed by 40 cycles of 95 ˚C for 15 s and 60 ˚C for 1 min. The 2-ΔΔCT method was used in the analysis of PCR data [34].

### Immunohistochemistry and histochemical stains

TS and ZEB1 immunohistochemical stains were performed in 71 PM biopsies using an automated platform (BenchMark, Ventana Medical Systems, Basel, Switzerland). Briefly, samples were pretreated for 36 min with antigen retrieval ULTRA CC1 then they were incubated for 40 min at 36° with TS (Ab 108,995, Clone EPR4545, 1:150 dilution, Abcam, Cambridge, UK) and ZEB1 (Ab HPA027524, polyclonal, 1:100 dilution, Sigma-Aldrich, St. Louis) primary antibodies. Both antibody staining scores were assessed by a pathologist (L.R.) using a semiquantitative histological score (H-score) as previously described [35].

BAP-1 (Ab sc-28383, clone C-4, 1:400, Santa Cruz, CA, USA) and p53 (Ab IR61661-2, clone DO-7, Ready-to-Use, Agilent Technologies, CA, US) immunostainings were performed using Dako Omnis System (Agilent Technologies, TX, US). BAP1 and p53 result interpretation was performed according to literature data [36, 37].

Silver impregnation stain kit (Diapath, Martinengo, Italy) was used for reticulin fibers detection according to the manufacturer’s protocol.

### Western blotting

Total proteins were obtained from cell culture utilizing RIPA lysis buffer (Thermo Fisher Waltham, Massachusetts, USA) supplemented with 1% protease and 1% phosphatase inhibitor cocktail (Complete; Roche, Basel, Switzerland). Protein concentration was determined using BCA protein assay kit (Pierce, Thermo Fischer, Waltham, Massachusetts, USA), and 30 μg of protein was resolved on a 10% SDS-PAGE gel and transferred to nitrocellulose membranes. Blots were blocked with 5% BSA in Tris-buffered saline-Tween 0.1% and incubated overnight at 4 °C with TS (1:1000 dilution, clone 106, Santa Cruz Dallas, Texas, USA) and Vinculin (1:1000; clone N19, Santa Cruz) antibodies. Immuno-reactive proteins were visualized using horseradish peroxidase-conjugated anti-mouse antibody. Proteins were detected by enhanced chemiluminescence substrate (Amersham, Little Chalfont, UK) and images acquired with Chemi-doc (Biorad Hercules, California, USA). The optical density of the appropriately sized bands was measured using the ImageJ free-software (http://rsbweb.nih.gov/ij), comparing each TS to control Vinculin band density.

### Cell transfection, treatment and viability assay

0.2 × 10^6^ cells were put into a 6-well plate one day before transfection. The following day, normal medium was replaced with a not complete one and the 682B, H226, REN, 672B, and 570B cell lines were transiently transfected with 25 pmol of miR-215, mir-375, and negative control mirVana miRNA mimic molecules (Life Technologies, Carlsbad, California, USA) using Lipofectamine RNAi MAX Reagent (Invitrogen, Carlsbad, California, USA). The day after, culture medium was replaced and after 48 h total proteins and RNA were collected.

miR-215 and miR-375 overexpression was confirmed by real-time PCR analysis on transfected cells and basing on results obtained we selected both transfected (t-) REN and t-570B cell lines as good transfection models. They showed the highest transfection efficiency and were representative of the epithelioid and sarcomatoid PM histotypes. Transfected-REN and t-570B were plated in triplicate into a 94-well plate. Then, they were treated with PEM (100 mg, Ely Lilly, Indianapolis, Indiana, USA) in different concentrations, as follows: Not Treated (NT), 0.01 uM, 0.1 uM, 1 uM, 10 uM, 100 uM (based on IC50 calculation). For each concentration, triplicate experiments were performed. After 48 and 72 h, 10 uL of WST-1 salt (Roche, Basel, Switzerland) was added to each well. Plates were incubated for 30 min at 37 °C before measuring the absorbance at 450 nm in a microplate reader (Biorad, Hercules, California, USA).

### Statistical analysis

Tissue and in vitro data were analyzed using the software GraphPad Prism version 5. In particular, Mann–Whitney, Student’ *t*, ANOVA, and Spearman tests were performed. A *p* value of less than 0.05 was considered statistically significant.

Overall survival (OS) was defined as the time from the date of diagnosis to the date of death or last follow up contact. Statistical significance (*p* < 0.05) of differences in OS between variables was tested using the log-rank test and visualized by the Kaplan–Meier curves.

## Results

### miRNAs expression in PM tissue samples

Patients’ characteristics were summarized in Supplementary Table 2. Seventy-one samples were retrieved, 4/71 (6%) of cases were biphasic, 7/71 (10%) sarcomatous, and 60/71 (84%) epithelioid PM.

**Fig. 1:**
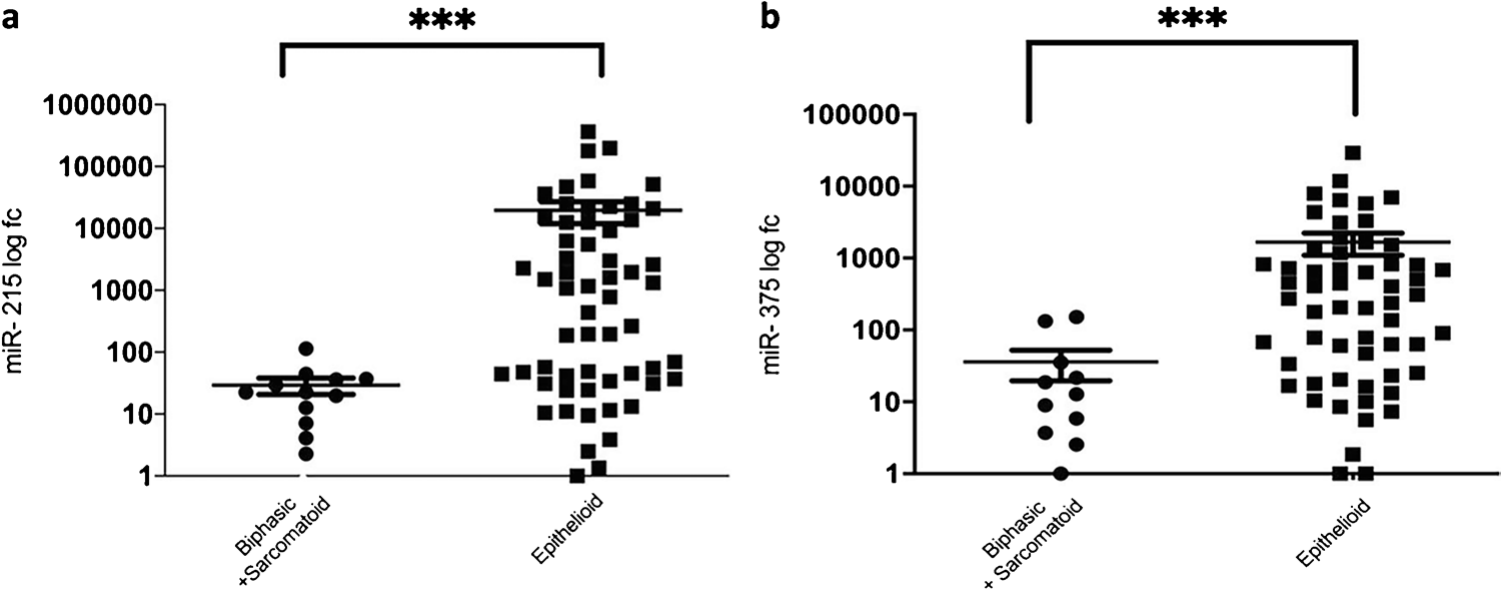
Representative dot plot analyses showing the different miR-215 and miR-375 expression in PM histotypes. (**a**) miR-215 showed higher statistically significant expression levels in epithelioid subgroup (*p* = 0.0002) than in the biphasic/sarcomatoid ones. (**b**) miR-375 showed higher statistically significant expression levels (*p* = 0.0005) in epithelioid than in biphasic/sarcomatoid cases. Each experiment was repeated in triplicate. All data in the figure were represented as mean logarithmic fold change ± SEM. fc, fold change; * *p* < 0.05, **p < 0.01, ***p < 0.001

**Fig. 2:**
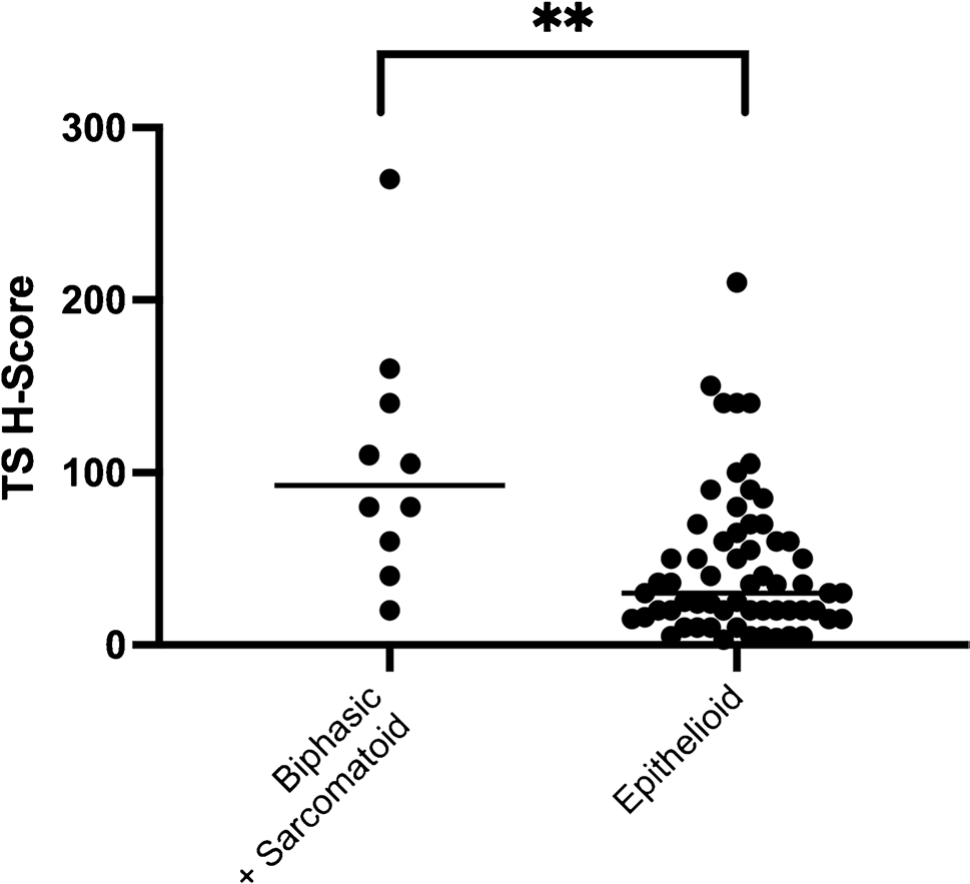
Representative dot plot analysis showing different TS H-Score protein expression in PM histotypes. H-Score, histological score. * *p* < 0.05, **p < 0.01, ***p < 0.001

Overall miR-215 and miR-375 were found heterogeneously expressed as well as TS immunohistochemical protein levels in PM tissues (Supplementary Fig. 1a and 1b, respectively). Comparison among histotypes showed significant over-expression of both miR-215 and miR-375 in epithelioid as compared to sarcomatoid and biphasic histotypes (Mann Whitney, *p* = 0.0002 for miR-215, *p* = 0.0005 for miR-375) (Fig. 1). On the contrary, TS protein levels showed higher expression in sarcomatoid and biphasic (H-score range 5–270, mean 100.83, median 92.5) as compared to epithelioid PM samples (H-score range 3–210, mean 45.9, median 30) (Mann Whitney *p* = 0.0013, Fig. 2). Furthermore, a strong direct correlation between miR-215 and miR-375 expression (Spearman test *p* < 0.0001, r = 0.71) was detected, and a significant opposite correlation between TS protein and both miRNA expression was found (Spearman test *p* = 0.009, r =  − 0.43 for miR-215 and *p* < 0.0001, r =  − 0.31 for miR-375). By contrast, no correlation was found between TS mRNA and both miRNAs (Table 1a). Similar results were obtained analyzing the epithelioid subgroup only: a significant direct correlation between miRNA-215 and miRNA-375 (*p* < 0.001) as well as a significant inverse correlation between TS protein (but not mRNA) and both miRNA-215 (Spearman *p* = 0.002, r =  − 0.40) and miRNA-375 (Spearman *p* = 0.01, r =  − 0.33) was found (Table 1b).

**Table 1:** Spearman test correlation of TS protein levels, miR-215 and miR375 expression with TS mRNA and miRNAs expression. a) overall series; b) epithelioid MPM only

a			
	**TS mRNA**	**miR-375**	**miR-215**
**TS protein**	r = 0.30	r = -0.31	r = -0.43
	***p* = *0.03***	***p* < *0.0001***	***p* = *0.009***
**miR-215**	r = 0.36	r = 0.71	-
	*p* = *n.s*	***p* < *0.0001***	-
**miR-375**	r = 0.18	-	-
	*p* = *n.s*	-	-
b			
	**TS mRNA**	**miR-375**	**miR-215**
**TS protein**	r = 0.3	r = -0.33	r = -0.40
	***p* = *0.02***	***p* = *0.01***	***p* = *0.02***
**miR-215**	r = -0.08	r = 0.73	-
	*p* = *n.s*	*p* = *n.s*	-
**miR-375**	r = 0.21	-	-
	*p* = *n.s*	-	-

**Footnotes**: TS: Thymidylate Synthase; PM: Pleural Mesothelioma.

### miR-215, miR-375, and TS mRNA and protein expression in PM cell lines

**Fig. 3:**
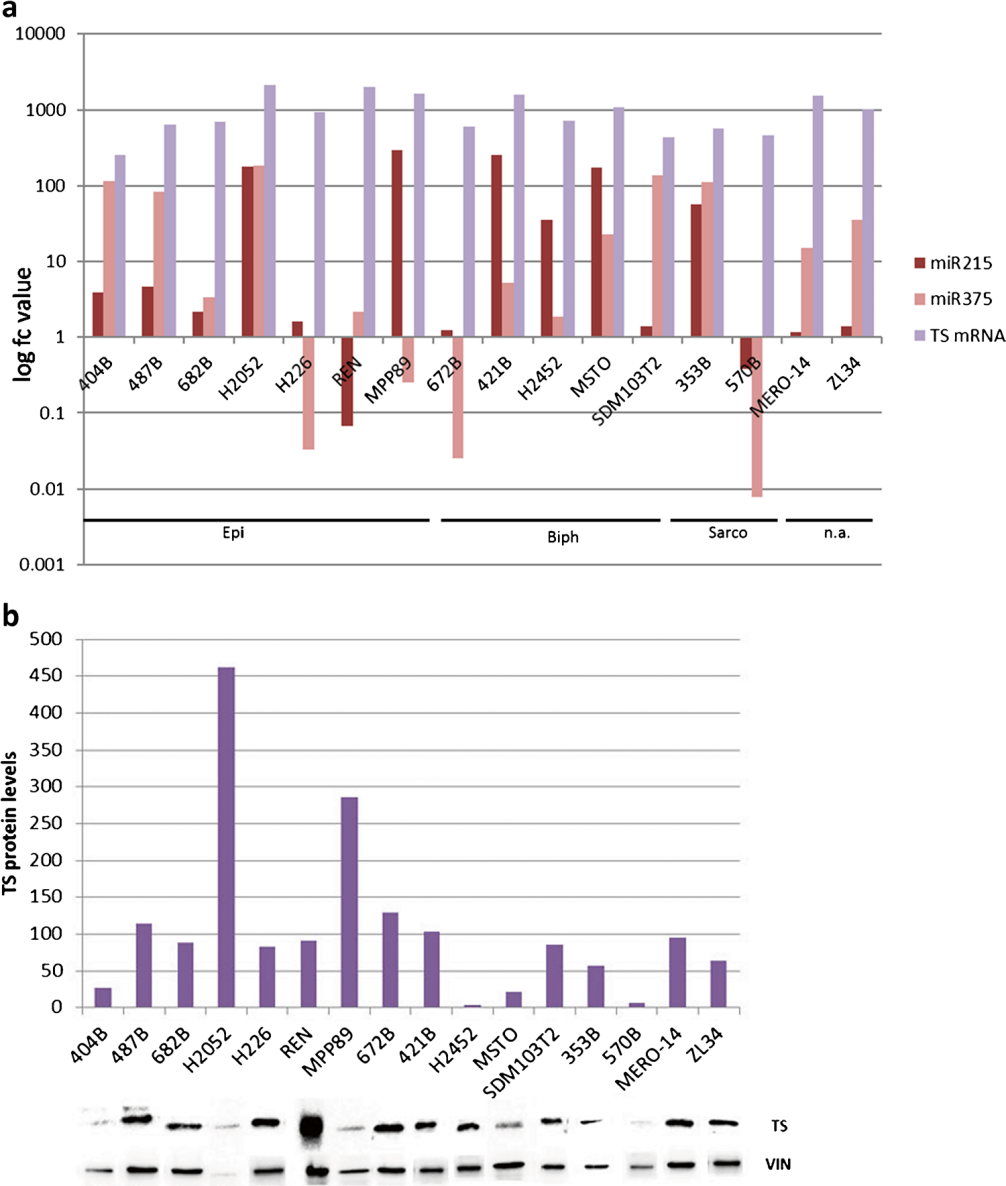
miR-215, miR-375, and TS (mRNA and protein) expression levels in 9 commercial and 7 patient-derived PM cell lines. (**a**) miR-215, miR-375, and TS mRNA expressions by real-time PCR analysis in PM cell lines. The graphic representation of the log fc value showed a heterogeneous distribution of miR-215, miR-375, and TS mRNA. (**b**) Graphic representation of TS protein expression detected by Western Blot analysis on 9 commercial and 7 patient-derived PM cell lines. A heterogeneous TS protein distribution was found. The Vinculin (VIN) expression was used as a control of equal protein loading. The optical density of the appropriately sized bands was measured using the ImageJ free-software http://rsbweb.nih.gov/ij, comparing each TS to control vinculin band density. TS, thymidylate synthase; VIN, Vinculin; EPI, epithelioid; BIPH, biphasic; SARCO, sarcomatoid. Each experiment was repeated in triplicate

A screening of miR-215, miR-375 and TS (mRNA and protein) expression levels was performed in 9 commercial and 7 patient-derived PM cell lines. Real-time PCR analysis revealed a heterogeneous miR-215, miR-375, and TS mRNA distribution (Fig. 3a). On the other hand, TS protein level measured by Western Blot (WB) analysis showed a higher expression of TS protein in the majority (12/16, 81%) of PM cell lines (except for 404, 570, H2452, MSTO cell lines) as compared to control Vinculin levels (Fig. 3b).

**Fig. 4:**
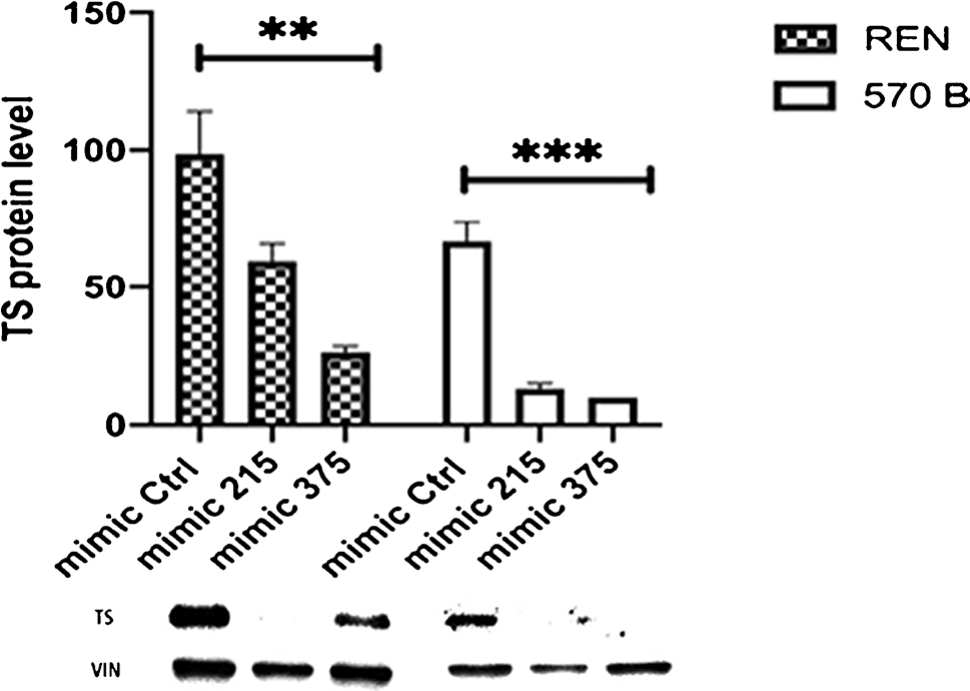
Western blot analysis on transfected REN and 570B cell lines. Western blot revealed lower expression levels of TS protein after mimic-miR-215 and mimic-miR-375 transient transfection compared with the REN and 570B cell lines transfected with the mimic-Ctrl. The experiment was repeated in triplicate and the data were represented as mean ± SEM. The optical density of the appropriately sized bands was measured using the ImageJ free-software http://rsbweb.nih.gov/ij, comparing each TS to control vinculin band density; TS, thymidylate synthase; VIN, Vinculin; Ctrl, control. * *p* < 0.05, **p < 0.01, ***p < 0.001

Based on these results, miR-215 and miR-375 transfection was performed on 682B, H226, REN, 672B, and 570 B cell lines. Only REN (epithelioid PM derived) and 570B (sarcomatoid PM derived) showed the highest transfection efficiency with miR-215 and miR-375 over-expression resulting in a strong reduction of TS protein expression levels (Fig. 4).

Then, we tested the role of miRNAs in modulating PEM sensitivity in vitro, by treating both the t-REN and t-570B PM cell lines with PEM at different timepoints and concentrations. Overall, not significant changes were recorded in cell viability assay with miR-215 and miR-375 transfected cells, in comparison to transfected mimic control. Only the t-REN, epithelioid PM cell line, showed a trend to a higher sensitivity to PEM, as compared to untreated t-REN cells, after 72 h of PEM treatment, although not reaching statistical significance. On the contrary, the miR-215 t-570B sarcomatoid cell line had a slight PEM-induced growth inhibition after 72 h of PEM treatment (Supplementary Fig. 2).

### miRNAs involvement in PM epithelial-to-mesenchymal transition

Given the higher expression levels of miR-215 and miR-375 in epithelioid than biphasic/sarcomatoid PM tissues, we investigated if they could have a role in mediating EMT by means of ZEB1 protein expression. ZEB1 immunohistochemical analysis on PM tissues revealed a heterogeneous expression and distribution in all PM biopsies (H-score values from 0 to 300, mean H-score value 156.6, median 150). ZEB1 expression was significantly higher in sarcomatoid (H-score value from 120 to 300, mean 246) as compared to epithelioid PM (H-score value from 0 to 300, mean H-score value 138) samples (Mann Whitney *p* = 0.0012) (data not shown). A significant direct correlation was found between both miRNAs and ZEB1 protein expression levels in epithelioid PM tissue samples (Spearman *p* = 0.01 for miR-215 and Spearman *p* = 0.009 for miR-375, respectively), while no overall correlation was found between TS and ZEB1 protein expression.

**Fig. 5:**
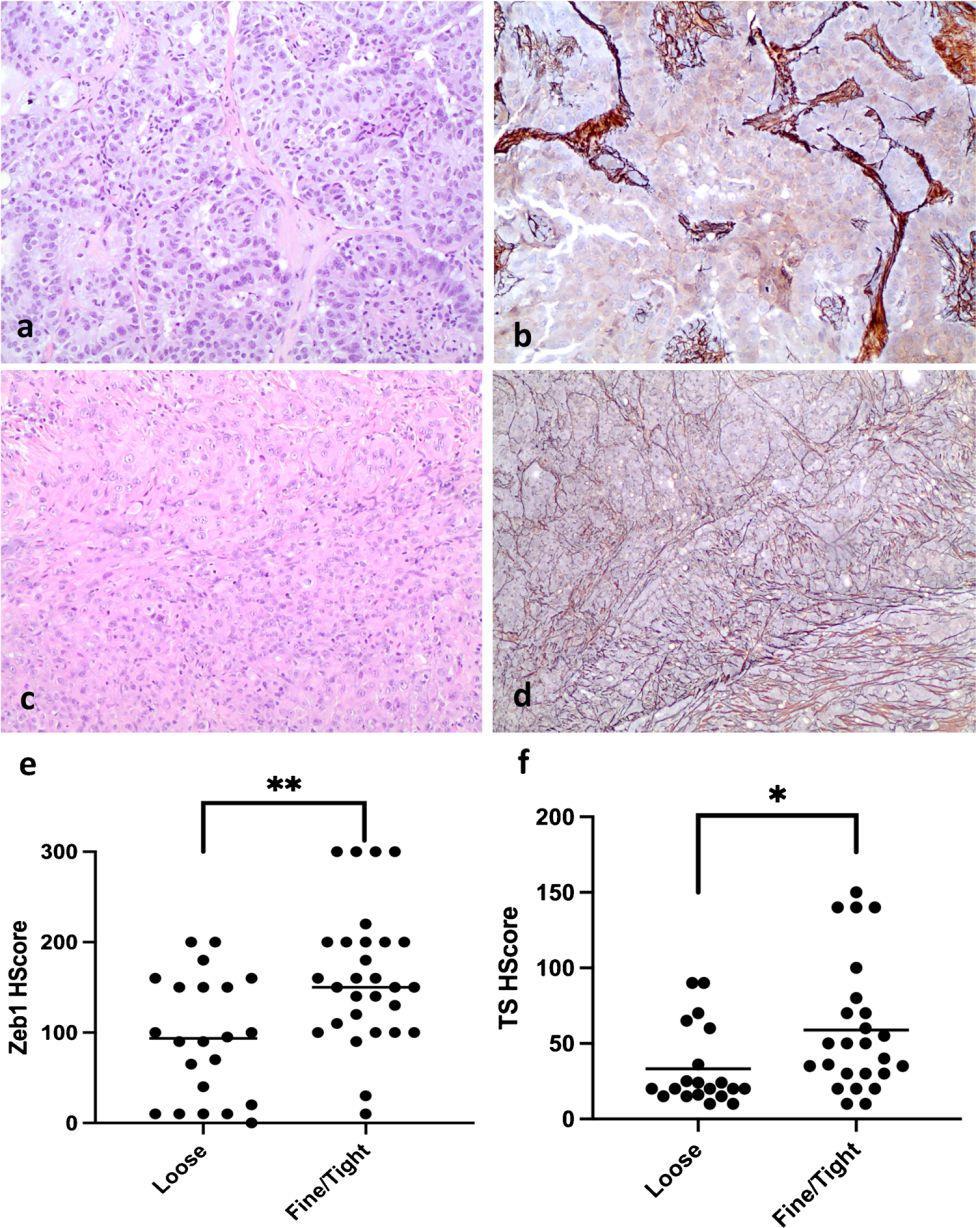
Reticulin pattern, ZEB1, and TS protein expression in epithelioid PM subgroup. (**a**) PM with clear epithelioid morphology (hematoxylin–eosin stain, 100 × magnification) and corresponding (**b**) reticulin fiber stain of the same case showing a loss of reticulin fibers surrounding very large tumor cell nests (loose-pattern) (silver reticulin stain, 100 × magnification); (**c**) PM with epithelioid morphology (hematoxylin–eosin stain, 100 × magnification) and corresponding (**d**) reticulin fiber stain of the same case showing a loss pattern of reticulin fibers surrounding large tumor cell nests (loose-pattern, upper left angle) associated to a fine, more filled reticulin pattern surrounding individual or small group of tumor cells (fine/tight pattern, lower right angle) (silver reticulin stain, 100 × magnification). Graphic representations of (**e**) ZEB1 and (**f**) TS protein expression according to loose or fine/tight reticulin pattern

Hence, we classified epithelioid PM tissues by means of reticulin stain to stratify different tumor growth patterns, according to the pattern of arrangement of collagen fibrils around tumor cells. As reported, sarcomatoid PMs had strong bands of coarse thickness narrow around individual cells (Tight, T-pattern) as compared to strapping of large cluster of epithelioid PM cells [38]. In our series, among the 51 epithelioid PMs available for reticulin stain, 22/51 (43%) had a pattern characterized by the reticular fiber framework lost or with loose meshes surrounding large tumor nests (Loose, L-pattern) (Fig. 5a, b). Interestingly, in 29/51 (57%) PM cases with the same epithelioid morphology, the L-pattern was associated to variable extension of tumor areas with denser, fine-to-tight reticulin fibers surrounding individual or small group of tumor cells (Fine/Tight pattern) (Fig. 5c, d). Moreover, considering the epithelioid PM subgroup only, we found that those cases with a component of Fine/Tight reticulin pattern, as compared to the epithelioid PM with a pure L-pattern, were characterized by significantly either higher ZEB1 protein expression levels (Mann–Whitney *p* = 0.002) (Fig. 5e), and significantly higher TS protein expression levels (Mann–Whitney *p* = 0.01) (Fig. 5f), and lower miR-215 and miR-375 expression levels, even if not significant (Mann–Whitney *p* = 0.25 and *p* = 0.08, respectively. Data not shown).

### miRNA and TS expression and morphology according to genetic subgroups.

Immunohistochemistry for BAP1 and p53 was performed in 62 cases (51 epithelioid, 4 biphasic and 7 sarcomatoid PM) with available material.

BAP1 nuclear expression was lost in 32/51 (63%) epithelioid, 1/4 (25%) biphasic and 1/7 (14%) sarcomatoid PM. No correlation was found between BAP1 alteration and miRNA expression, nor TS expression nor different epithelioid morphology (data not shown).

p53 complete loss or overexpression (altered status) was found in 7/51 (14%) epithelioid, 0/4 biphasic and 4/7 (57%) sarcomatoid (3 completely lost in tumor cells with positive internal control and 1 overexpressed). No correlation was found between altered p53 and miRNA expression, nor TS expression nor different epithelioid morphology (data not shown).

### Survival analyses

Follow-up was complete in 57/71 (80%) of patients from 1 to 52 months. At the time of the study, 60/71 (85%) patients were died. Median OS was 10 months.

A little significant difference was found in survival curves between cases with low and high TS protein expression: lower levels of TS protein give a slightly better survival as compared to higher levels (12 vs. 8.5 months, log-rank *p* = 0.05). No significance for survival was found between cases with low or high expression of miRNA-215 and miRNA-375 or between cases with epithelial PMs with loose or fine-to-tight reticulin stain (Supplementary Fig. 3).

**Fig. 6:**
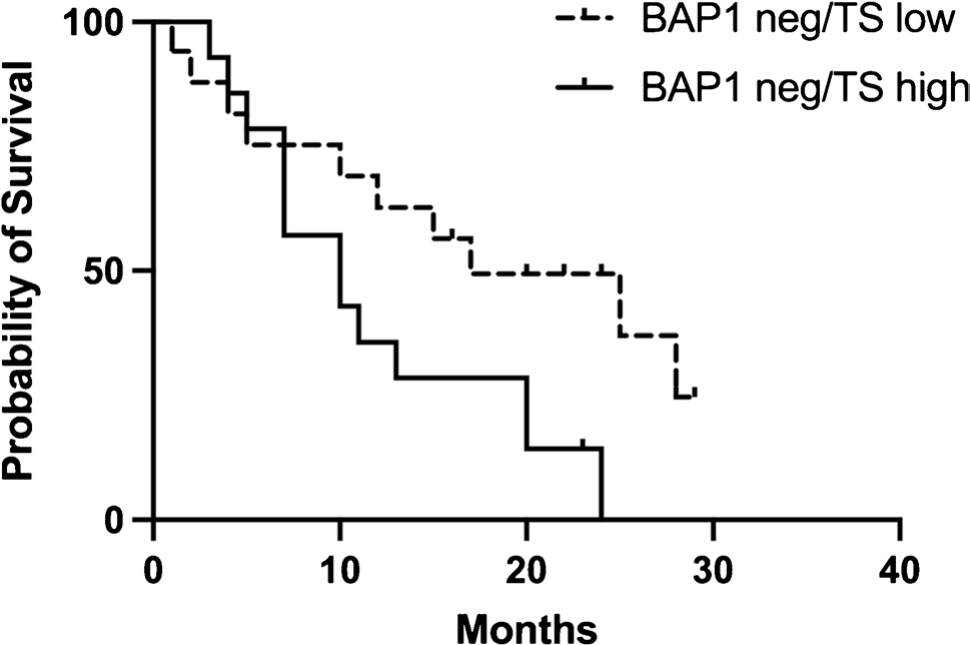
Survival curves of cases according to BAP1 status and TS protein levels. A significant better survival was found for those cases with BAP1 loss and TS low protein levels (median survival 17 vs. 10 months, log-rank *p* = 0.03)

As regard genetic profile, BAP1 status showed a scarce correlation (log-rank *p* = 0.09, data not shown) with survival while no significance was found for p53 and survival. However, considering BAP1 negative cases only, no significance for survival was found between the high or low expression of miRNA-215 and -375 or between epithelial PMs with or without EMT morphology (data not shown); on the contrary, a significant better survival was found for those cases with BAP1 loss and TS low expression (17 vs. 10 months, log-rank *p* = 0.04) (Fig. 6), thus confirming the central role of TS protein in regulating PM progression.

## Discussion

In this study, we demonstrated that (1) miR-215 and miR-375 are expressed in PM tissues in a significant histotype-dependent manner and inversely correlated with TS protein levels; (2) miR-215 and miR-375 induce a specific modulation on TS protein expression in PM cell lines, although this not implied changes in sensitivity to PEM in vitro; and (3) the regulatory activity of miR-215 and miR-375 could be involved into EMT in PM tissues via TS protein expression modulation.

Despite the recent improvements in targeted oncology therapy, no efficient option is still available for PM treatment, a part of recent advances in immunotherapy [39, 40]. One of the causes of therapeutic failure in treating PM is due to the development of resistance to standard chemotherapy, namely PEM-based, largely attributed to the increased expression of the TS enzyme, the main target of PEM [41]. Our group demonstrated that immunohistochemical levels of TS could be predictive of response to therapy in a selected series of PEM-treated PM patients. According to our study, patients with higher levels of TS tumor protein demonstrated poorer overall survival, while a low-to moderate TS level showed a better correlation with PEM-based response to therapy [42]. However, few evidence demonstrated a possible modulation strategy of TS both on PM and other cancers.

Micro-RNA-215 and -375 were recently found to target TS in other tumors [23], so, due to the important predictive role of TS in PM, we explored their potential role in targeting and modulating TS in PM. Micro-RNAs were already studied as modulator of TS expression in several cancer models [43], and specific miRNAs were described in PM diagnosis and prognosis [44, 45], but scanty evidences about therapeutic role of miRNAs were described in PM [46]. In particular, only mir-215-5p was recently reported to exerted significant cell killing by activating p53 function and inducing apoptosis [24]. On the other hand, at the best of our knowledge, no functional evidence on mir-375 were reported in PM.

In our consecutive series of PM tissues, the gene expression of miR-215 and miR-375 revealed a significant specific distribution of both miRNAs according to PM main histotypes: both miRNAs were more highly expressed in the better epithelioid than in the poorer biphasic and sarcomatoid histotypes, thus suggesting a correlation of the miRNA expression levels with a different program of neoplastic mesothelial cells differentiation. This result confirmed literature data by Siddiqui and coworkers who correlated miR-215 and miR-375 lower expression with less aggressive form of tumor and described them as negative modulators of EMT phenomenon [23, 47]. In the present series, TS protein levels were found significantly higher in biphasic and sarcomatoid than in epithelioid PM (Mann–Whitney *p* = 0.0013). This observation is not surprising given the role of TS in tumor cell cycle, the poorer histotype-related prognosis in PM and the high chemotherapy resistance of mesenchymal histotypes. Interestingly, TS protein and both miR-215 (*p* = 0.009) and miR-375 (*p* < 0.0001) expressions were overall significantly inversely correlated in the present series and a strong correlation between both miR-215 and miR-375 expression (Spearman test *p* < 0.0001, r = 0.71) was also found, in line with the hypothesis of a same target for these two miRNAs to TS.

Based on these results, we decide to induce a transient overexpression of miR-215 and miR-375 in PM cell lines for evaluating their activity on TS modulation in vitro: both transfected cell lines showed a direct decrease of TS protein expression, thus confirming that TS is a target of miR-215 and miR-375 and supporting the significant inverse correlation between miRNAs and TS protein expression found in PM tissues. Unfortunately, viability assay failed to reveal a significant growth inhibition in PEM-treated miRNAs overexpressing cells, respect to untreated control. In a previous study, Abu Lila et al. obtained an improvement of chemosensitivity to PEM in vitro and in vivo reducing TS expression by shRNA transfection in PM models [11]. This data was not confirmed by our study, but a different experimental model was used. Moreover, TS gene silencing in a tumor cell could reduce the tumor cell proliferation per se, for the cell cycle role of TS in tumor cells. Our aim was to modulate TS, as we obtained with the transient transfection, but, besides TS modulation, other complex signaling pathways could take part on regulating cell growth response to PEM.

Different reasons could be at the base of this event. Firstly, it is known that PEM has multiple targets: other than TS, it could also inhibit dihydrofolate reductase (DHFR), and glycinamide ribonucleotide transformylase (GART) enzyme activity [5], so its inhibitory effect on PM patients could be due also to other secondary mechanisms. Secondly, the capability of cancer cells to escape the drug toxicity could depend on several complex mechanisms other than TS protein action [48, 49].

Finally, to better investigate the role of miRNA in PM and clarify their different expression distribution in PM histotypes, particularly in epithelioid PM, we investigated a possible involvement of miR-215 and miR-375 into PM EMT process. Published data demonstrated that ZEB1 is one of the hallmark of EMT [29] and one of its activity is related to repress miR-375 [23]. We investigated ZEB1 immunohistochemical expression in PM tissues and we found that ZEB1 was significantly (Mann Whitney, *p* = 0.0012) overexpressed in the sarcomatoid histotype (H-score mean value: 245.9) compared to the epithelioid PM (H-score mean value 138.11) confirming already published data [33]. However, in our tissue series, no correlation between miRNA and ZEB1 protein expression was found, thus suggesting that epithelial or mesenchymal differentiation could be regulated not by ZEB1 activity on these miRNAs in PM. Interestingly, we found a variable distribution of the ZEB1 levels in epithelioid PM subgroup ranging from undetectable to high levels. To test if this distribution could represent a different propensity of higher ZEB1-expressing epithelioid PM cases to progress to a mesenchymal histotype, we classified PM morphological features by means of reticulin stain, to better define different histological component. Recently, specific reticulin patterns were described in different PM histotypes [50], with a loose pattern associated to epithelioid and a tight pattern to sarcomatoid histotype. In our series, the pure epithelioid subgroup was characterized by a completely loose pattern of reticulin fibers, but in a half of epithelioid PM cases, reticulin stain highlighted the presence of a tumor component with fine-to-tight reticulin pattern associated to the pure epithelioid. We hypothesized that this feature could represent an initial transformation to a more mesenchymal type, probably representing cases with initial “transformed” patterns [51]. Interestingly, in this group, both higher protein levels of ZEB1 and TS expression were found. Recently, Siddiqui et al. [23, 47] described a role of TS, other than ZEB1, to drive EMT in non-small cell lung cancer, with direct correlation between the two molecules. In our PM series nor direct correlation between TS and ZEB1 was found, neither inverse correlation between miRNAs and ZEB1. On the contrary, those epithelioid PM with a fine-to-tight reticulin pattern associated component were characterized by a significantly higher TS levels and miR-375 and miR-215 lower expression levels, confirming these miRNAs as strong modulator of TS in EMT phenomenon [23].

Furthermore, we segregate our cases from a genetic point of view into groups according to BAP1 and TP53 altered or wild-type status, since these two genes are the most mutated in PM [14] and their mutational status could be proved by immunohistochemistry with reliable results [36, 37]. According to these groups, we found no significant correlation neither in miRNA or TS expression, nor in PM with or without EMT morphology. These events lead us to conclude that neither BAP1 nor TP53 genetic profile influence the expression and function of miRNAs in targeting TS. Furthermore, in our series, BAP1 loss was not associated with EMT morphology of epithelioid PM, as already reported by de Reynies and coworkers that found the mesenchymal phenotype more associate with a BAP1 retained status [52].

Taken together, our results allowed us to hypothesize that TS could take part in the epithelial to mesenchymal transition in PM, providing an additional reason for the poorer outcome of high TS expressing cases, linked not only to an intrinsic chemoresistance [42], but also to a propensity towards a more aggressive histotype. Nonetheless, it must be noticed that PM is per se a unique biological model of complex tumor characterized by the epithelial and mesenchymal type but also by the coexistence of both epithelial and mesenchymal features in the same tumor sample, suggesting an intrinsic epithelial-to-mesenchimal transition [53].

Finally, we found a borderline significance (*p* = 0.05) for survival analysis in those cases with different TS protein levels, while we failed to found significant survival differences either for miRNA expression, or for EMT morphology. Nevertheless, the curve trends seem to suggest that the state of miRNAs high or TS low or EMT absent confers a better patient outcome.

Furthermore, we found a significant better survival for those cases with both BAP1 loss and TS low protein levels, thus confirming the central role of TS modulation in PM patients’ outcome (Fig. 6).

A limit of these analyses is the consecutive and retrospective nature of our series that implies the heterogeneity of clinical patient stages at diagnosis and the different therapeutic treatments received that could have influenced the survival. Nevertheless, even if we are aware that this data do not demonstrate a direct effect of miRNAs in patient better survival, our study suggests that the modulation/inhibition of TS protein levels by miRNAs could be a good strategy to prevent PM growth.

## Conclusions

In conclusion, in the present study, we demonstrated that TS could be a target of both miR-215 and miR-375 in PM tumor model; TS modulation by these miRNAs is not associated with response to PEM nor to EMT-marker ZEB1; however, the strong association of both miRNAs and TS expression with PM histotypes and — in epithelioid group — with mesenchymal-like component, suggests that this pathway might drive EMT processes in epithelioid PM. Furthermore, at least in BAP1 mutated patients, low TS protein levels are associated with better patients’ survival.

## Supplementary Information

Below is the link to the electronic supplementary material.

Supplementary file1 (DOCX 14254 KB)

## Data Availability

Data and material are available.
