# Correction to: Micro-RNA-215 and -375 regulate thymidylate synthase protein expression in pleural mesothelioma and mediate epithelial to mesenchymal transition

**DOI:** 10.1007/s00428-022-03355-y

**Published:** 2022-06-07

**Authors:** Francesca Napoli, Ida Rapa, Stefania Izzo, Angelica Rigutto, Roberta Libener, Chiara Riganti, Paolo Bironzo, Riccardo Taulli, Mauro Papotti, Marco Volante, Giorgio Scagliotti, Luisella Righi

**Affiliations:** 1 Department of Oncology, University of Turin, San Luigi Hospital, Regione Gonzole 10, Orbassano, Turin, Italy; 2 Pathology Unit, San Luigi Hospital, Orbassano, Turin, Italy; 3 Present Address: Department of Medical Oncology and Hematology, University Hospital of Zurich, Zurich, Switzerland; 4 Pathology Division, Saints Antonio and Biagio Hospital, Alessandria, Italy; 5 Interdepartmental Research Center of Molecular Biotechnology, University of Turin, Turin, Italy; 6 Interdepartmental Centre for Studies On Asbestos and Other Toxic Particulates, University of Turin, Turin, Italy; 7 Center for Experimental Research and Medical Studies (CeRMS), City of Health and Science University Hospital, Turin, Italy; 8 Pathology Unit, City of Health and Science University Hospital, Turin, Italy

**Correction to: Virchows Archiv**

10.1007/s00428-022-03321-8

In the original published version of the article above contain incorrect affiliation of the authors Angelica Rigutto and Luisella Righi. The below affiliations are presented ad corrected as follows:

