# Supplementary material for: Micro-RNA-215 and -375 regulate thymidylate synthase protein expression in pleural mesothelioma and mediate epithelial to mesenchymal transition
Source: Virchows Arch. 2022 Apr 24;481(2):233–44. doi: 10.1007/s00428-022-03321-8 (PMC9343276; doi:10.1007/s00428-022-03321-8)
Supplement: Supplementary file 1 — Supplementary file1 (DOCX 14254 KB) [file 428_2022_3321_MOESM1_ESM.docx]

**Supplementary Materials**

**Supplementary Table 1:** ~~M~~PM cell lines characteristics.

| **CELL LINES** | **DERIVATION** | **MPM HISTOTYPE** |
| --- | --- | --- |
| 404B | patient derived | epithelioid |
| 487B | patient derived | epithelioid |
| 682B | patient derived | epithelioid |
| H2052 | commercial cell line | epithelioid |
| H2452 | commercial cell line | epithelioid |
| H226 | commercial cell line | epithelioid |
| REN | commercial cell line | epithelioid |
| MPP89 | commercial cell line | epithelioid |
| 672B | patient derived | biphasic |
| 421B | patient derived | biphasic |
| MSTO | commercial cell line | biphasic |
| SDM103T2 | commercial cell line | biphasic |
| 353B | patient derived | sarcomatoid |
| 570B | patient derived | sarcomatoid |
| MERO-14 | commercial cell line | n.a. |
| ZL34 | commercial cell line | n.a. |
|  |  |  |
|  |  |  |

Notes: n.a.: not available

**Supplementary Table 2**. clinical and pathological parameters of 71 ~~M~~PM patients with miRNAs and TS expression

| **~~MPM~~ HISTOTYPE** | **SEX** | **MEAN AGE** | **TS mRNA (log fc median value)** | **TS HScore (median value)** | **miR-215**  **(log fc median value)** | **miR-375**  **(log fc median value)** |
| --- | --- | --- | --- | --- | --- | --- |
| **Biphasic + Sarcomatoid** (#11) | 11 males  0 female | 71  (55-81) | 0.89 | 80 | 22.5 | 12.8 |
| **Epithelioid** (#60) | 38 males  22 females | 72  (46-88) | 0.85 | 32.5 | 1073.69 | 238.06 |

**Supplementary Figure 1**. (**a**) Graphic representation of the log fc value of miR-215 and miR-375 in 71 ~~M~~PM patients obtained by means of Real Time PCR analysis. A heterogeneous miR-215 and miR-375 distribution was found. The experiment was repeated in triplicate. (**b)** Graphic representation of TS protein levels detected by IHC staining on 71 ~~M~~PM patients. A heterogeneous TS protein distribution was found. All the patients were grouped according to their different ~~M~~PM histotype: epithelioid (Epi), biphasic (Biph) and sarcomatoid (Sarco).


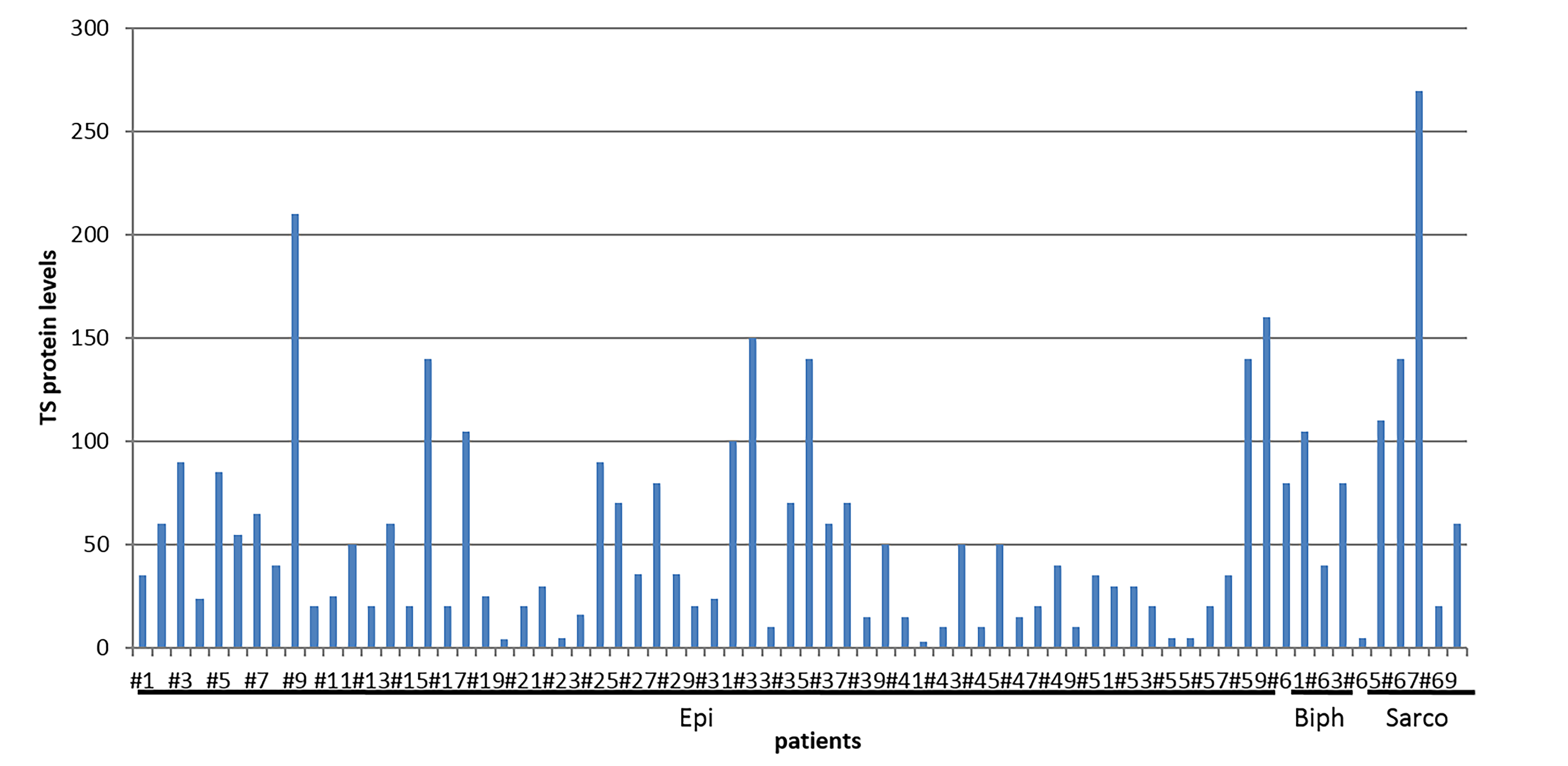

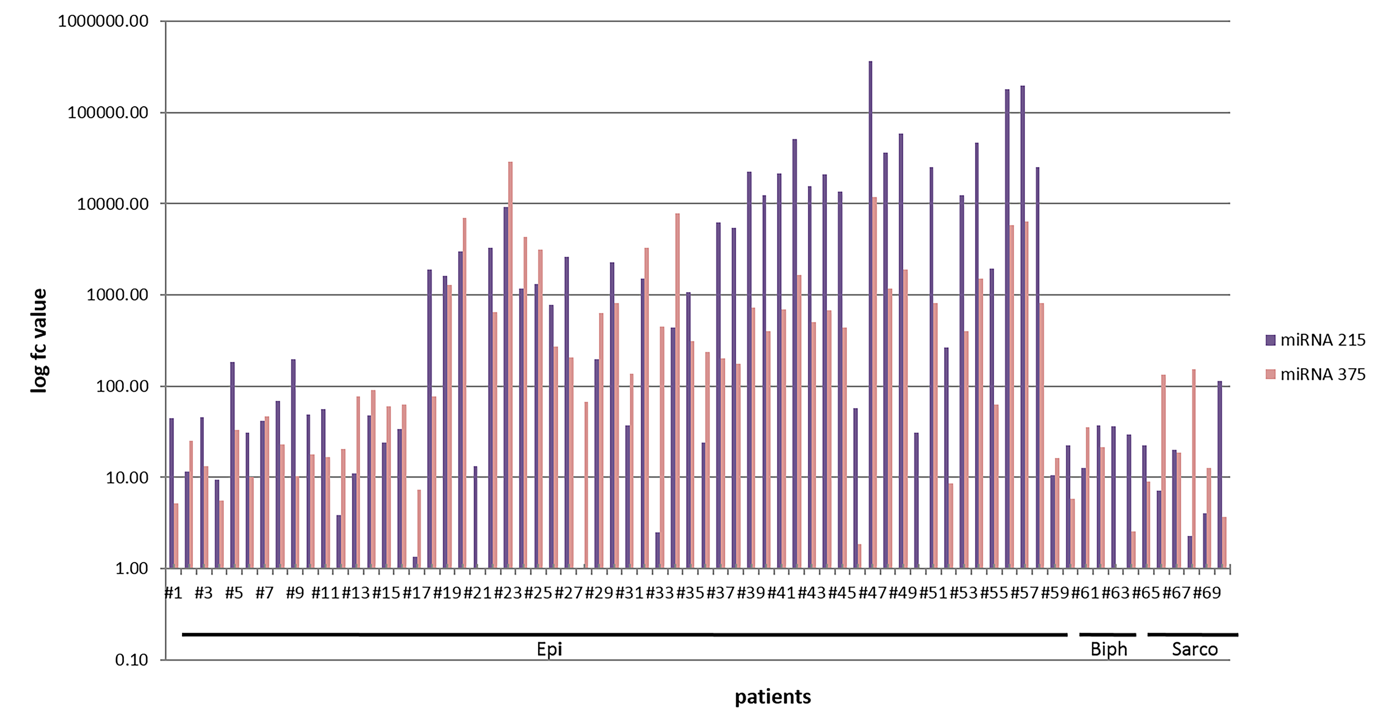


**Supplementary Figure 2.** Viability assay curves of mir-215, mir-375 and mir-Ctrl transfected cell lines at different timepoints and PEM concentration a) Viability assay curves of mir-215, mir-375 and mir-Ctrl t-REN after 48 hours. b) Viability assay curves of mir-215, mir-375 and mir-Ctrl t-REN after 72 hours c) Viability assay curves of mir-215, mir-375 and mir-Ctrl t-570B after 48 hours. d) Viability assay curves of mir-215, mir-375 and mir-Ctrl t-570B after 72 hours. Figure were represented as mean ± SD. Notes: NT= not treated; ctrl: cells transfected with control.


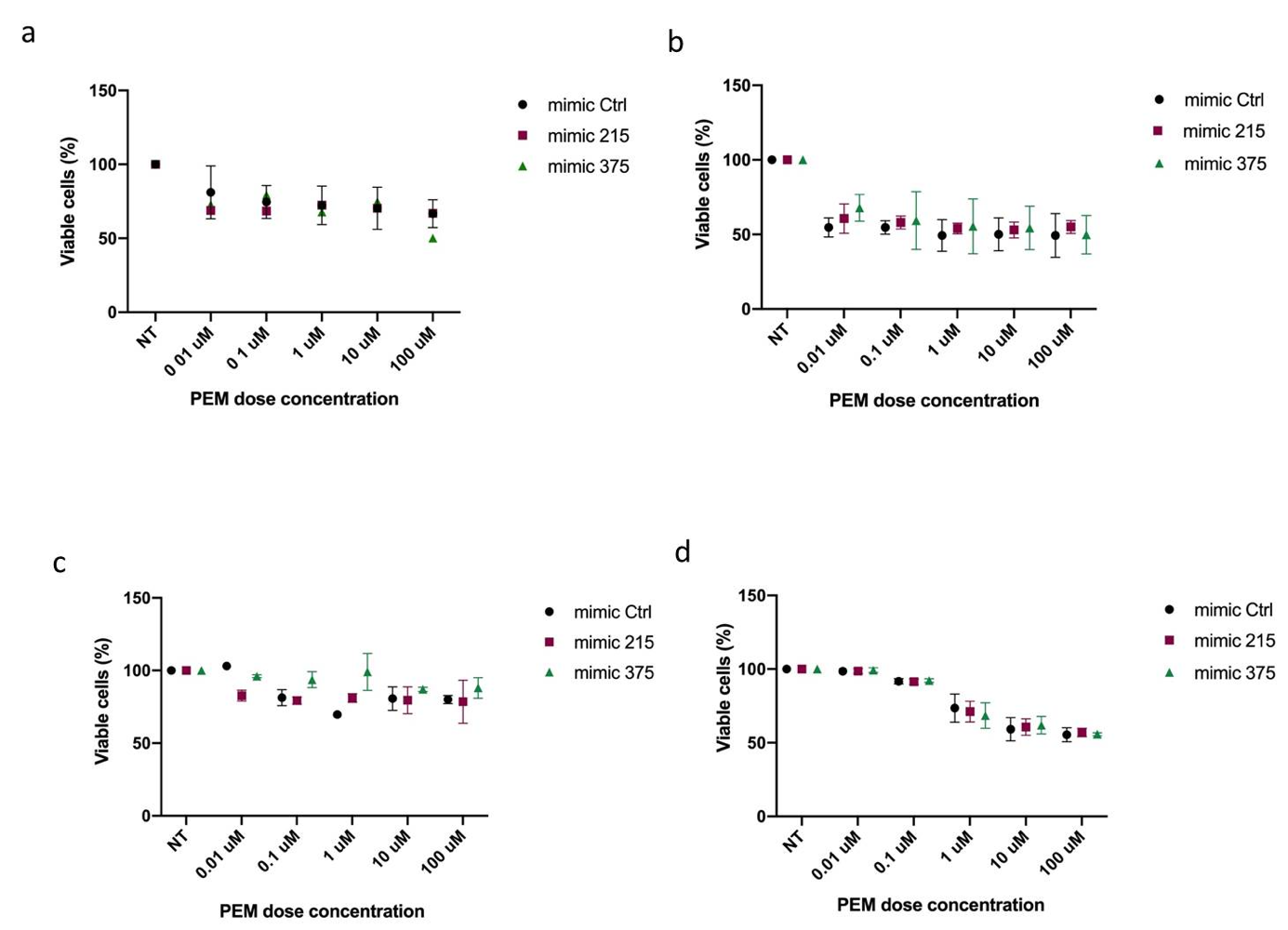


**Supplementary Figure 3**

Kaplan-Meir survival curves of cases with available follow up data. **a)** A little significance was found in survival curves between cases with low and high TS expression: lower levels of TS protein expression showed a slightly better survival (12 vs 8.5 months, log-rank p=0.05) with respect higher levels. No significance for survival was found between cases with low or high **b)** miRNA-215 and **c)** miRNA-375 expression (log-rank, p=0.3 and p=0.2, respectively) or between **d)** epithelial ~~M~~PMs with loose or fine-to-tight reticulin stain (log-rank p=0.09).

**
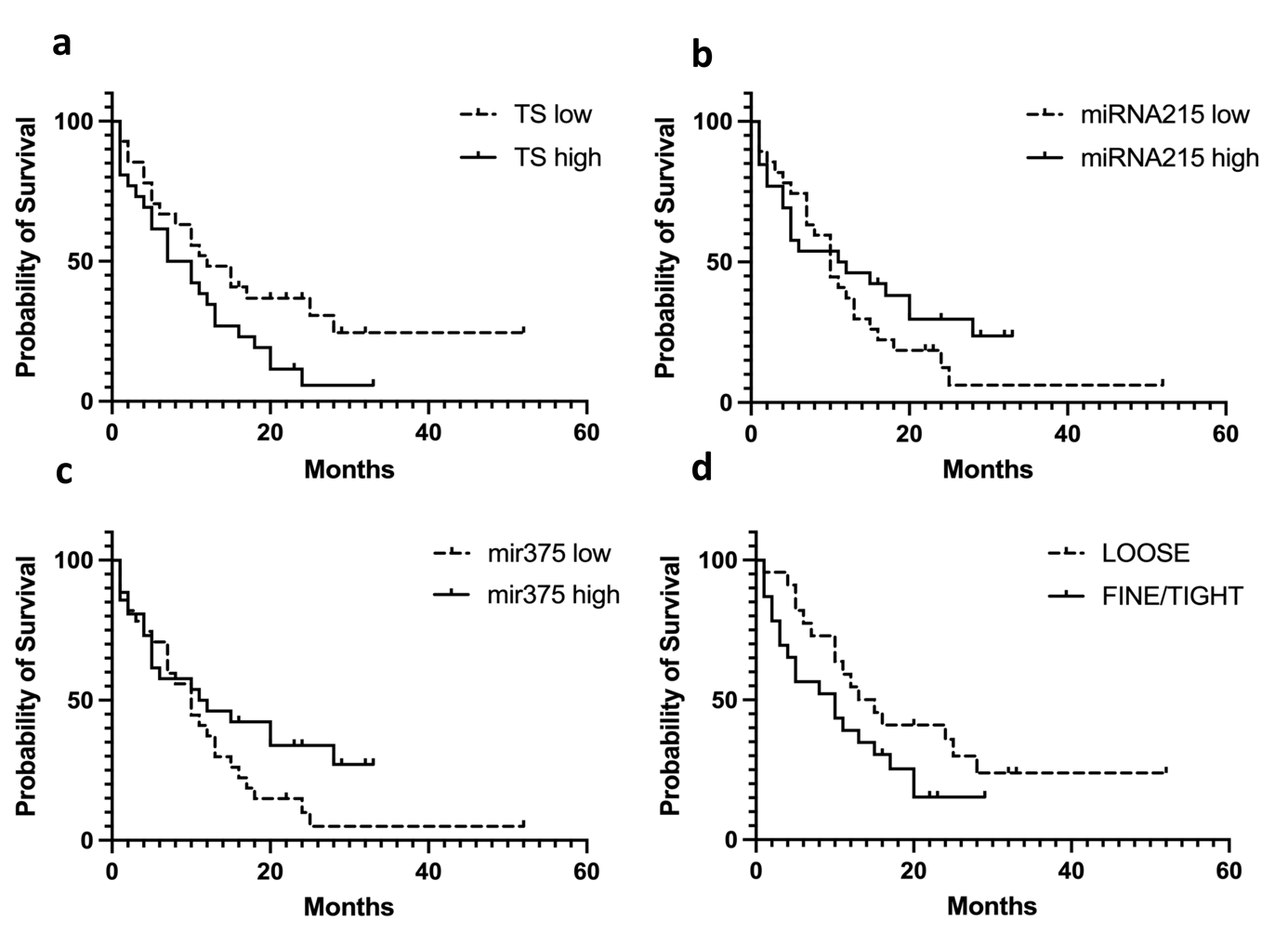
**
